# SARS-CoV-2 omicron spike simulations: broad antibody escape, weakened ACE2 binding, and modest furin cleavage

**DOI:** 10.1128/spectrum.01213-22

**Published:** 2023-08-31

**Authors:** M. Zaki Jawaid, A. Baidya, R. Mahboubi-Ardakani, Richard L. Davis, Daniel L. Cox

**Affiliations:** 1 Department of Physics and Astronomy, University of California, Davis, California, USA; 2 Protein Architects Corp, Penn Valley, Pennsylvania, USA; Memorial Sloan Kettering Cancer Center, New York, New York, USA

**Keywords:** SARS-CoV-2, omicron, spike, ACE2, furin, antibody escape

## Abstract

The recent emergence of the omicron variant of the SARS-CoV-2 virus with large numbers of mutations has raised concern about a potential new surge in infections. Here we use molecular dynamics to study the biophysics of the interface of the BA1 and BA2 omicron spike protein binding to (i) the ACE2 receptor protein, (ii) antibodies from all known binding regions, and (iii) the furin binding domain. Our simulations suggest that while there is a significant reduction of antibody (Ab) binding strength corresponding to escape, the omicron spikes pay a cost in terms of weaker receptor binding as measured by interfacial hydrogen bonds (H-bond). The furin cleavage domain (FCD) is the same or weaker binding than the delta variant, suggesting lower fusogenicity resulting in less viral load and disease intensity than the delta variant.

**IMPORTANCE:**

The BA1 and BA2 and closely related BA2.12.2 and BA.5 omicron variants of SARS-CoV-2 dominate the current global infection landscape. Given the high number of mutations, particularly those which will lead to antibody escape, it is important to establish accurate methods that can guide developing health policy responses that identify at a fundamental level whether omicron and its variants are more threatening than its predecessors, especially delta. The importance of our work is to demonstrate that simple *in silico* simulations can predict biochemical binding details of the omicron spike protein that have epidemiological consequences, especially for binding to the cells and for fusing the viral membrane with the cells. In each case, we predicted weaker binding of the omicron spike, which agreed with subsequent experimental results. Future virology experiments will be needed to test these predictions further.

## INTRODUCTION

The omicron variant of the SARS-CoV-2 virus was first detected publicly in November 2021 (1) and traced back to variants which appeared in mid-2020. Because the variant contains a large number of mutations relative to the original strain, including three relevant regions of the viral surface spike protein [the receptor binding domain (RBD), the furin cleavage domain (FCD), and the N-terminal domain (NTD)], the variant is of great concern. According to current GISAID data, the global infection landscape is almost exclusively dominated by omicron sub-variants, particularly BA1, BA2, and BA2.12.1, with recent emergence of BA4 and BA5 (2).

The fitness of a particular variant depends upon several factors. First, strong binding to surface receptors is of critical importance, and the SARS-CoV-2 RBD binds with high affinity to the angiotensin converting enzyme 2 (ACE2) protein in human cells (3). This contrasts with likely weaker binding of coronaviruses associated with the common cold such as OC43 that binds more weakly to sialic acid groups on the cell (4). Second, escaping the background antibody (Ab) spectrum can confer relative fitness over the dominant variant. Third, efficient membrane fusion and transmission are apparently strongly regulated by the FCD, where cleavage can arise both by furin and by transmembrane serine proteases, especially TMPRSS2 (5). It has been shown, for example, that ferrets inoculated with a WT SARS-CoV-2 with the FCD deleted can become infected but fail to transmit to other ferrets (6). The delta variant in cultured cells containing endogenous levels of ACE2 and TMPRSS2 has shown significantly enhanced fusion of the viral membrane with the cell membrane (5). The high viral load of the delta variant has been clearly associated with the mutation P681R of the FCD (7) and has led to the current dominance of SARS-CoV-2 sequences worldwide prior to the omicron emergence (8).

Given the time lag in carrying out protein synthesis, structure determination of bound complexes, determining protein binding affinities, and measuring viral neutralization by Abs for new variants, there is clearly a role for rapid computational studies that can assess the differences of new variants relative to background variants as they arise.

In this paper, we point out here that computational *ab initio* molecular dynamics studies of omicron subvariants RBD-ACE2, RBD-antibody (AB), FCD-Furin, and NTD-antibody are consistent with (i) robust antibody escape in all regions compared to wild type (WT) and delta, (ii) FCD binding to furin intermediate between WT and delta, and (iii) weaker binding to the ACE2 than WT or delta. The Ab escape can confer transmissibility advantages for a population with a prevalent delta variant Ab spectrum, but the weaker binding to ACE2 and modest enhancement of furin binding are likely to lead to weaker transmissibility than delta. Due to the high degree of similarity in the RBD and NTD regions of the BA2, BA2.12.1, BA4, and BA5 variants, we present simulation results and subsequent comparisons for WT, delta, BA1, and BA2 variants. For reference, the BA2 RBD is identical to BA2.12.2 RBD with the exception of one mutation (L452Q), and the BA4 and BA5 RBD with the exception of residues 486 and 493. The NTD of BA2 and BA2.12.2 are identical, while BA4 and BA5 NTD have an additional couple of deletions compared to BA2 NTD. The FCD for all the aforementioned omicron variants is identical.

At the time of writing, the current global infection landscape is dominated by BA2 (24%), BA2.12.2 (13%), BA4 (4%), and BA5 (38%) (2). This work uses ColabFold’s (9) implementation of AlphaFold-Multimer (10) to generate structures for FCD-Furin binding.

## MATERIALS AND METHODS

### Molecular models

A summary of all the mutations in the RBD and N-terminus of the spike protein for the four variants presented here is found in Table S1.

We drew our starting structures for RBD-ACE2 binding from the PDB file (11). For Class I ABs, which bind in the same region of the RBD as the ACE2, we used C1A.B12 [PDB:7CJF (12)] and 7KVF P4A1 [PDB:7KVF (13)] (P4A1), while as a representative class III Ab that binds to the RBD away from the ACE2 interface, we used CR.3022 [PDB:6YOR (14)]. For an NTD-Ab we used 4A8 [PDB:7C2L (15)].

**Fig 1:**
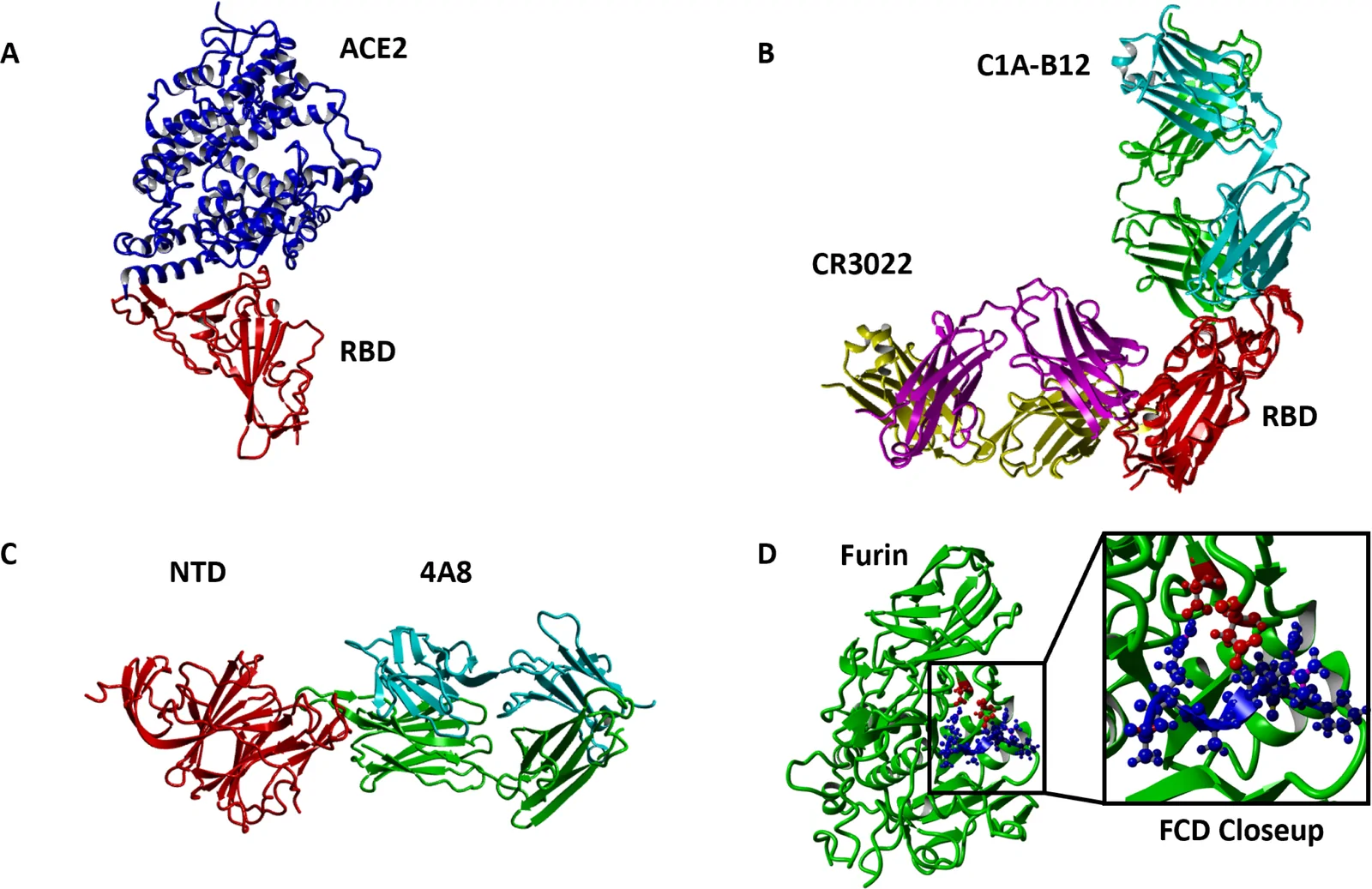
Structures of WT spike protein complexes studied (A) ACE2 (red)-RBD (blue) binding (PDB 6m0J). (B) Binding of RBD (red) to Class I Ab C1A-B12 (binds in ACE2 interface region; heavy chain green, light chain cyan, PDB 7KFV) and Class III Ab CR3022 (binds away from ACE2; heavy chain magenta, light chain yellow, PDB 6YOR). (C) Binding of NTD to 4A8 Ab (heavy chain green, light chain cyan, PDB 7C2L). (D) Binding of FCD (blue) to furin (red). Blowup highlighting the position of fifth residue R5 (R685 for WT SARS-CoV-2) with proximate aspartic acid residues D151 and D199 of the furin enzyme. All AlphaFold PDB files are provided in the Supplementary Material.

The antibodies chosen do not comprehensively portray all neutralizing Abs for the SARS-CoV-2 spike but are representative of the spectrum of antibodies that neutralize the SARS-CoV-2 virus. This study does not account for t-cell binding sites (16). Figure 1 shows the structures of the different complexes studied in this paper.

### Molecular dynamics

To simulate the protein-protein interactions, we used the molecular-modeling package YASARA (17) to substitute individual residues and to search for minimum-energy conformations on the resulting modified structures of the complexes listed in Table S2 (hydrogen bonds) and Table S3 (binding energy estimates). For all of the structures, we carried out an energy-minimization (EM) routine, which includes the steepest descent and simulated annealing (until free energy stabilizes to within 50 J/mol) minimization to remove clashes. All molecular-dynamics simulations were run using the AMBER14 force field with (18) for solute, GAFF2 (19) and AM1BCC (20, 21) for ligands, and TIP3P for water. The cutoff was 8 Å for Van der Waals forces [AMBER’s default value (22)] and no cutoff was applied for electrostatic forces [using the Particle Mesh Ewald algorithm (23)]. The equations of motion were integrated with multiple timesteps of 1.25 fs for bonded interactions and 2.5 fs for non-bonded interactions at T=298 K and P=1 atm (NPT ensemble) via algorithms described in (24). Prior to counting the hydrogen bonds and calculating the free energy, we carry out several pre-processing steps on the structure including an optimization of the hydrogen-bonding network (25) to increase the solute stability and a $p\,K_{\text{a}}$ prediction to fine-tune the protonation states of protein residues at the chosen pH of 7.4 (24). Insertions and mutations were carried out using YASARA’s BuildLoop and SwapRes commands (24), respectively. Simulation data were collected every 100ps after 1–2 ns of equilibration time, as determined by the solute root mean square deviations (RMSDs) from the starting structure. For all bound structures, we ran for at least 10 ns postequilibrium and verified the stability of time series for hydrogen bond counts and root mean square deviation from the starting structures. Because of concerns about the validity of short time simulations and more variability for the weaker binding for the omicron RBD-ACE2 complexes, we ran for 30 ns postequilibration in those cases.

The hydrogen bond (H-bond) counts were tabulated using a distance and angle approximation between donor and acceptor atoms as described in reference (25).

Note that in this approach, salt bridges of proximate residues are effectively counted as Hbonds between basic side chain amide groups and acidic side chain carboxyl groups.

We provide all molecular dynamics simulation analysis, including PDB snapshots, RMSD/F, as well specific residue-residue H-bond interactions for all 24 of our simulations in the supplemental material. Net hydrogen bond counts are summarized in Supplemental Table

### Endpoint free energy analysis

We calculated binding free energy for the energy-minimized structure using the molecular mechanics/generalized Born surface area (MM/GBSA) method (26 – 28), which is implemented by the HawkDock server (29). While the MM/GBSA approximations overestimate the magnitude of binding free energy relative to *in vitro* methods, the obtained values correlate well with Hbond counts. For each RBD-ACE2, RBD-AB, and NTD-Ab binding pair we average over 10 snapshots of equilibrium conformations. For each FCD-Furin pair, we average over five snapshots of equilibrium conformations.

### Use of colabfold/alphafold for furin cleavage domain

Due to the absence of structural data for the FCD-Furin bound complex, we model the FCD-Furin bound structure using the heterocomplex prediction method known as AlphaFold-Multimer (10, 30) as implemented within ColabFold (9) to predict the best bound structure to the furin enzyme of the six residue FCD from the WT protein. We inferred the ordering of this sequence by comparison with a very similar six residue peptide inhibitor of furin with the sequence RRRVR-aminomethyl-benzamidine (RRRVR-amba) (31). In this case, the backbone of the WT FCD aligns well with that of the inhibitor, but the fifth arginine enters a furin pocket while the amba enters the furin pocket for the inhibitor. The serine is in proper cleavage position for furin. The delta and omicron structures were then obtained by mutation from the predicted WT FCD-Furin structure. In a separate work, we present a complete description of the use of ColabFold/AlphaFold for modeling the FCD-Furin binding as well as simulation results of over 60 observed FCD sequences for SARS-CoV-2 and other commonly observed coronaviruses (32). In this study, we limit our FCD-Furin binding focus to sequences from WT, delta, and omicron variants.

All PDB files generated using AlphaFold as well as the simulated data associated with them are provided in the supplemental material.

### Statistical analysis

We computed the statistical significance of pairwise differences using GraphPad unpaired *t*-test.

## RESULTS

### Binding strengths: H-bond and binding free energy

Before discussing our results, it is important to contextualize what a single H-bond difference makes. From earlier work, it has been estimated that a single H-bond in a beta sheet is stabilized by 1.6 kcal/mol (33). At room temperature (*RT* = 0.59 kcal/mol), therefore, using this as a baseline estimate, we would reduce $K_{D}$ by a factor of exp⁡(1.6/.59)≈14 for a single bond, and, e.g., in the case of the four H-bond difference for furin binding of the delta FCD over the WT FCD, we would have a reduction of $K_{D}$ by $\approx 5\times 10^{4}$ , clearly much stronger binding. We are intending the use of these numbers only for characterizing the significance of the H-bond count for energetics and affinity, not to be taken as quantitatively accurate estimates since H-bond energetics depend sensitively upon context.

**Fig 2:**
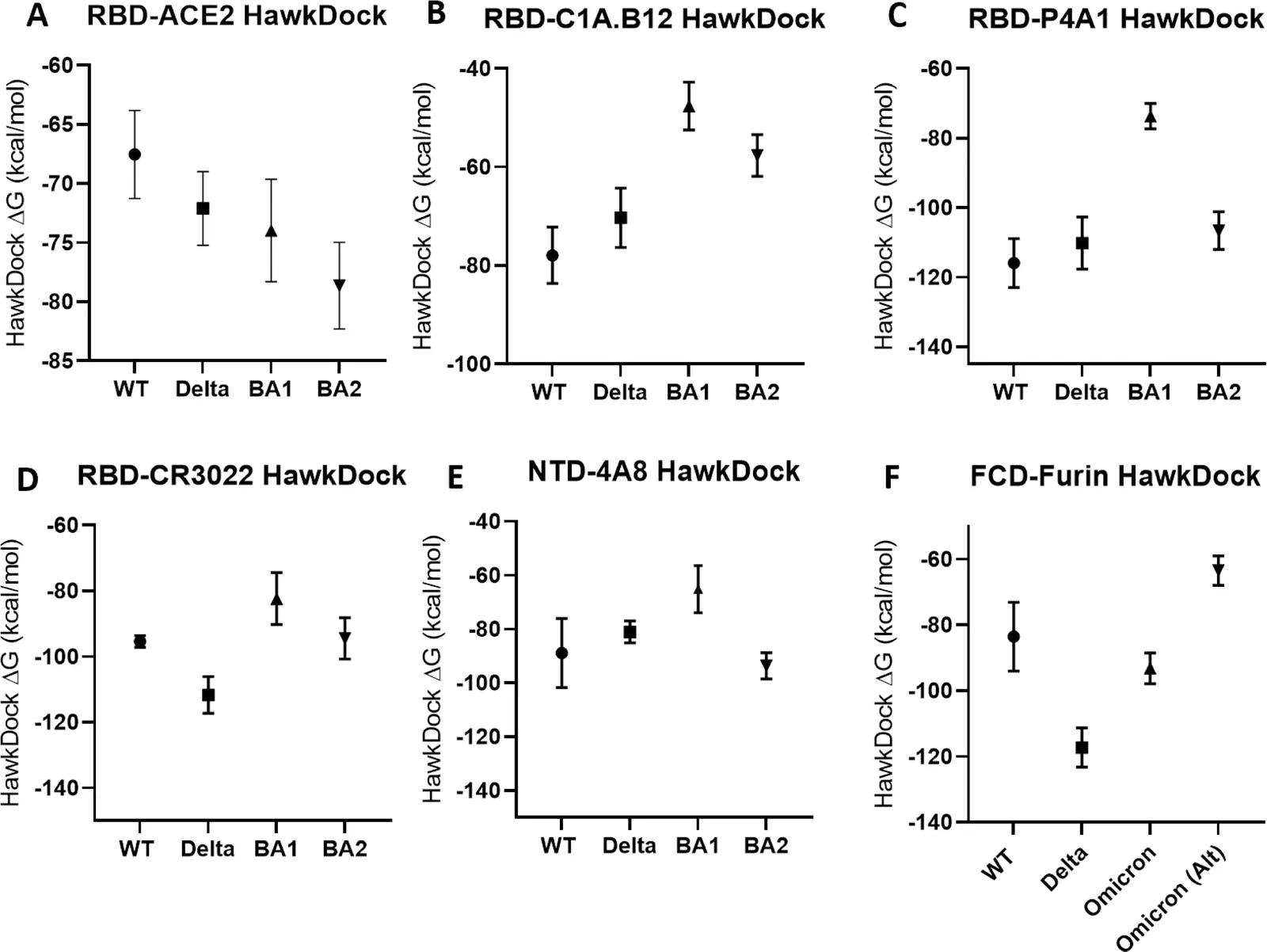
Interfacial hydrogen bonds between proteins for WT, delta, BA1, and BA2. All bars represent 95% CI. (A–E) BA1 and BA2 variants participate in significantly fewer interactions than WT and Delta for the simulations shown, with the exception of Delta and BA2 in (E). All pairwise *P*-values in Fig. 2A through E are *P* < 0.0001 (highly significant), with the exception of the aforementioned Delta versus BA2 pair in (E) (*P* = 0.19, not significant), the BA1 versus BA2 pair in (B) (*P* = 0.82, not significant), the WT versus delta pair in (C) (*P* =0.09, not significant), and the BA1 versus BA2 pair in (D) (*P* = 0.01, significant). (F) FCD-Furin H-bond interactions. All omicron variants participate in slightly higher interactions than WT but less than Delta. We also consider the possibility of the FCD for the omicron variants starting at 679K in Omicron (Alt). All pairwise *P*-values in (F) are *P* < 0.001 (highly significant). All PDB files are referenced in the methods section and provided in the repository referenced in the supplemental material.

Our main results for interfacial H-bonds for the structures of Fig. 1 are summarized in Fig. 2. We find somewhat weaker binding to the ACE2 receptor compared to both WT and delta, which should moderate infectivity, and significant antibody escape of the BA1 and BA2 for all three regions (Class I, Class III, and NTD) considered, with the exception of RBD-P4A1 binding for BA2 compared to WT (but still weaker than delta). This escape is measured by the reduction in hydrogen bond count between the antibodies and the spike protein.

For the FCD-Furin binding, six residues fit into the binding pocket, which we argue elsewhere to begin with residue 681 for WT, alpha, and delta (32). For omicron, we consider the possibility of leading with the N679K mutation or P681H mutation and denote N679K leading furing binding as “Omicron Alt” in Fig. 2. The P681H mutation leading is the same as the alpha variant. We see that the expected binding to the FCD is at best the same as the alpha variant and significantly less than the delta variant.

For the omicron RBD-ACE2 runs, as alluded to above, we carried out additional simulation time for 30 ns versus 10 ns, and we found significantly decreased variability for the last 20 ns. In comparison with WT, for both the full 30 ns and the last 20 ns the *P*-value is smaller than 0.0001 indicating extreme statistical significance.

For differences between measured H-bond counts, we provide all *P*-value pairs in Fig. 2, together with 95% confidence intervals.

**Fig 3:**
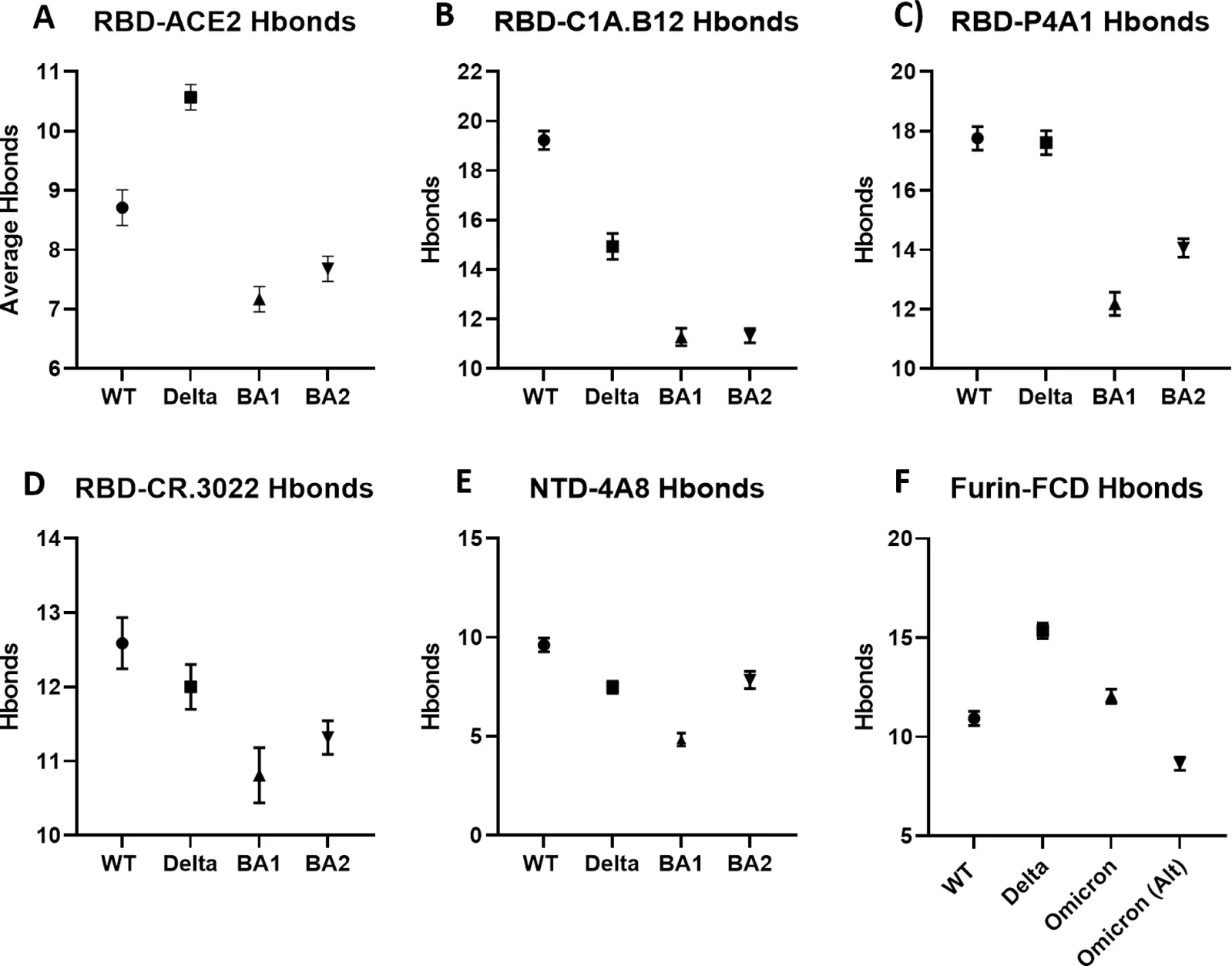
GBSA Binding free energy estimate in kcal/mol between proteins for WT, delta, BA1, and BA2. All bars represent 95% CI. (**A–F**) MM/GBSA binding free energy estimates correlate strongly with the number of H-bonds in Fig. 2 with the exception of the RBD-ACE2 interactions. All PDB files are referenced in the methods section and provided in the Supplementary Material. *P*-values for all pairs in (A–F) are <0.001 with the following exceptions: (A) WT versus Delta (*P* = 0.053, not significant), WT versus BA1 (*P* = 0.022, significant), Delta versus BA1 (*P* = 0.44, not significant), Delta versus BA2 (*P* = 0.007, significant), and BA1 versus BA2 (*P* = 0.08, not significant). (B) WT versus Delta (*P* = 0.053, not significant), Delta versus BA2 (*P* = 0.0013, significant), BA1 versus BA2 (*P* = 0.0024, significant). (C) WT versus Delta (*P* = 0.096, not significant), WT versus BA2 (*P* = 0.0036, significant), Delta versus BA2 (*P* = 0.23, not significant). (D) WT versus BA1 (*P* = 0.0049, significant), WT versus BA2 (*P* = 0.75, not significant). (E) WT versus Delta (*P* = 0.21, not significant), WT versus BA1 (*P* = 0.0033, significant), WT versus BA2 (*P* = 0.44, not significant), and Delta versus BA1 (*P* = 0.0026, significant).

The binding energies from the GBSA analysis of molecular dynamics equilibrium conformations are shown in Fig. 3. The same PDBs are utilized. Evidently the trend of binding energies tracks well with the easier to estimate interfacial H-bond count, with the exceptions of the ACE2-omicron RBD binding.

However, it is very clear that the confidence intervals in Fig. 3 are relatively larger and overlap more than those from Fig. 2. The primary reason is the number of measurements. Because we can draw on large numbers (order hundreds) of simulation snapshots to analyze H-bond counts, the error bars are smaller than for GBSA calculations for which time constraints have allowed only 10 snapshots for each interface.

### Mutations leading to Ab escape and weaker ACE2 binding

**Fig 4:**
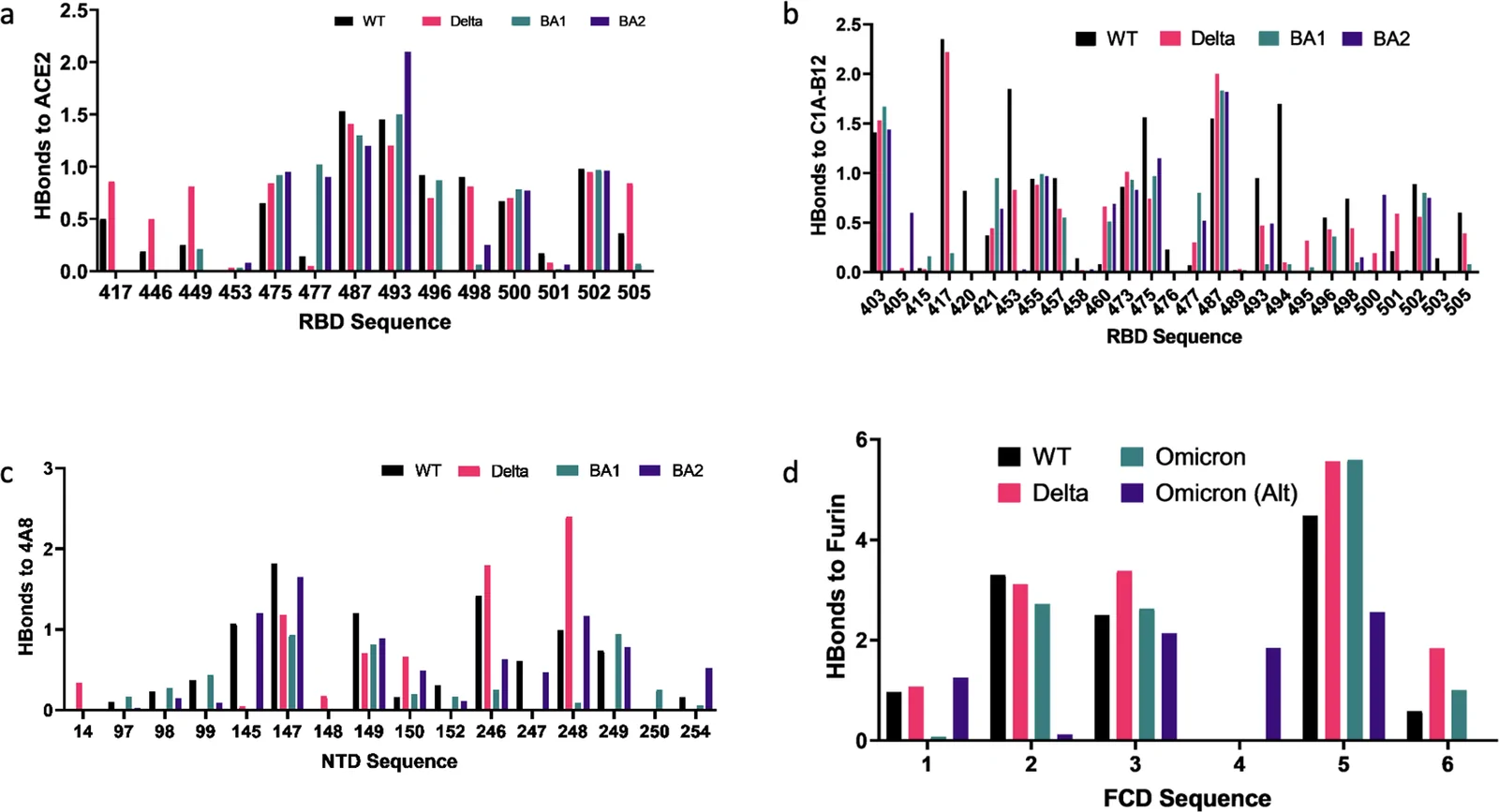
Overview of binding changes for delta and omicron variants relative to WT Color coding is the same for all charts. For the FCD to furin binding, R1-R6 correspond to 681–686, except for the alternate omicron sequence 679–684. For clarity, RBD binding to P4A1 and CR3022 Abs are not shown. Full residue interaction tables measured by average hydrogen bound strengths are provided in the Supplementary Material.

Figure 4 illustrates the key mutations leading to differences in binding for the delta and omicron variants relative to WT.

For ACE2 binding, these mutations weaken the ACE2 binding for omicron relative to WT: (i) K417N removes the K417(RBD)-D30(ACE2) salt bridge. (ii) Q498R removes hydrogen bonding between the glutamine side chain and K353 of the ACE2 driven by R-K Coulomb repulsion. (iii) Y505H removes hydrogen bonding between the Y505 sidechain and the E37 sidechain of ACE2 where the Y505 O acts as a donor. On the other hand, the S477N mutation of omicron enhances bonding relative to wild type, the Q493R mutation enhances the binding to the E35 and D38 acidic residues of ACE2, and the N501Y mutation enhances binding relative to WT. As discussed, the net effect is a reduced number of interfacial hydrogen bonds overall. A qualitative picture is provided in Fig. 4A, while numerical values for detected residue pairs are provided in Table S4 to S7.

For Class I antibodies, the following mutations are critical for reducing the binding strength of omicron: For binding to P4A1, (i) the Y455 binding to Y33.HC of the Ab heavy chain (HC) is removed. (ii) The Q493K, G496S, and Q498R mutations lead to removal of bonds with E101.HC, W32.LC of the Ab light chain, and S67.LC. iii) The Y505H mutation removes bonds to S93.LC. For binding to C1A-B12, (i) the K417N mutation removes a salt bridge to D96.HC, a side chain bond to S98.HC, and weakens a side chain bond to Y52.HC. (ii) The mutations Q493R, G496S, and Q498R remove bonds to R100.HC, S30.HC, and S67.HC. (iii) The N501Y and Y505H mutations weaken bonds in the 501–505 region to G28.LC, S30.LC, and S93.LC. A complete list of detected residue pairs is provided in Table S8 to S15. Figure 4B shows binding changes relative to WT for C1A-B12.

For the Class III antibody CR3022, the most noticeable differences compared to WT are (i) the absence of binding at N370 to Y27.HC. This appears to be driven by the hydrophobic substitution S371L, which pulls the asparagine at 370 out of bonding distance from Y27.HC. (ii) Weakened bonding of T385 to S100.HC. A complete list of detected residue pairs is provided in Table S16 to S19.

For the NTD Ab 4A8, we find that the notable differences of omicron compared to WT are (i) weakened binding at 145–152 presumably due to the deletion at 142–145 relative to WT and (ii) significantly weakened bonding at 246–254 driven by the EPE insertion at 214 and the deletion at 211. Both the 142–145 deletion and the 211 deletion with EPE insertion disrupt the epitope positionings at 145–152 and 246–254, respectively. A complete list of detected residue pairs is provided in Table S20 to S23. Figure 4C shows binding changes relative to WT for Ab 4A8.

### Mutations in the FCD

The FCD (also known as the S1/S2 cleavage site) of SARS-CoV-2 differs from that of SARS-CoV-1 by a polybasic insertion beginning at P681 (34). Successful cleavage of this region by the Furin enzyme is associated with increased cell-to-cell and viral transmission *in vitro* (35). Furthermore, the polybasic insertion at the FCD has been shown to confer SARS-CoV-2 with a selective advantage in lung cells and primary human airway epithelial cells (6).

Due to the absence of structural data for the FCD as well as the FCD-Furin bound complex, there are limited computational studies of the binding domain. This is because the FCD belongs to a rapidly fluctuating random coil region of the protein that has not been resolved by structural probes [see, e.g., Ref (36), PDB structure 7A94, for which residues 677–688 are unresolved]. Additionally, there are no bound Furin-FCD structures available due to furin rapidly cleaving the protein at this domain.

For the generic 681–686 sequence of the FCD, our simulations show that the most critical residue appears to be the 685. In the WT, the arginine is able to form a salt bridge in the interior pocket with D199 of the furin and bond additionally with S146, W147, D151, A185, and S261. This tendency is illustrated in Fig. 4D. These bonds are all strengthened for delta and omicron. For the alternate KSHRRA sequence of the omicron, beginning at 679, the position of the arginine in the binding pocket allows only the salt bridge formation with D199. The FCD sequences for the omicron subvariants BA1, BA2, BA2.12.1, BA4, and BA5 are identical and are therefore not differentiated for this part of the study.

**Fig 5:**
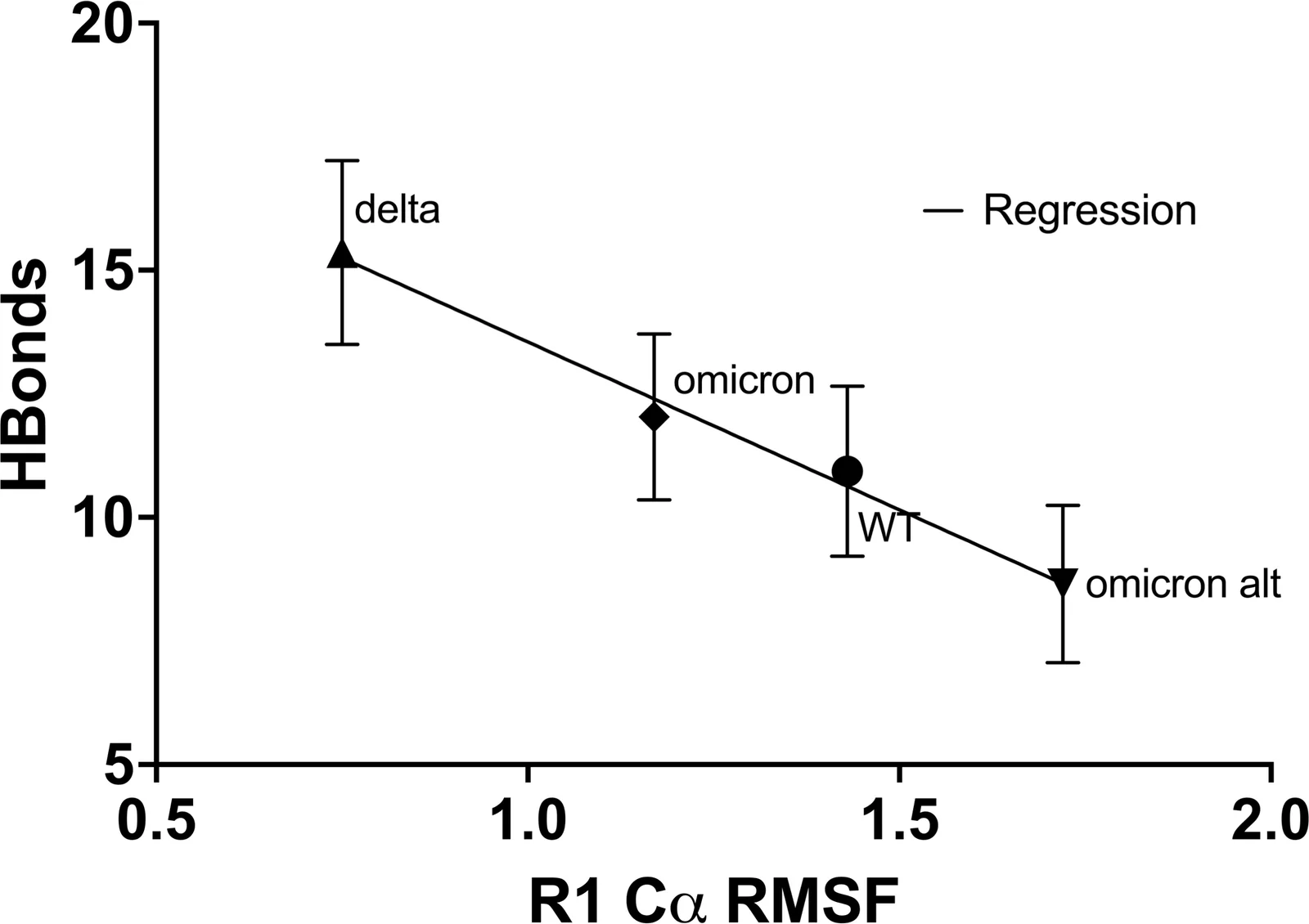
Correlation of FCD-Furin interfacial H-bond count with RMSF of the first residue in FCD. The higher the RMSF of the first residue in the FCD, the harder it is to bind to the furin, especially for the critical fifth residue which inserts into the furin pocket as shown in Fig. 1D. R1 is residue 681 for all but the alternate omicron sequence which starts at residue 79. Full simulation data (RMSF/D) is provided in the repository link of the Supplemental Material. All *P*-values for H-bond counts between pairs are reported in Fig. 2. The equation of the regression line is *Hbonds* = −6.7 ± 0.7(*RMSF*) + 20.1 ± 0.9, with regression coefficient *R* ^2^ = 0.98 and is probably negative with *P* = 0.01 (significant).

As shown in Fig. 5, we observe that the binding strength, which is determined to a large degree by the binding of the fifth residue of the FCD, correlates inversely with the root mean square fluctuation (RMSF) of the backbone C α of the first FCD residue at 681. This suggests that locking the 681 C α as happens for P681R is a key to lowering the fluctuation spectrum of the 685 residue allowing for stronger binding at this site. Evidently, the gain in binding enthalpy offsets any advantages in conformational entropy for the FCD.

In a separate work, we test the FCD-Furin binding for over 80 observed and unobserved sequences (32). We find that among all candidate viral sequences studied, the delta is near the very top binding strength within statistical accuracy. The binding strength of several rare sequences matches delta with statistical accuracy as well as some unobserved sequences. Of these, we find that the sequences resulting from P681K (KRRARS) or P681S (SRRARS) mutations in the FCD could, in theory, match delta’s binding strength for the FCD-Furin binding. All current omicron variants (BA1–BA5) have P681H (2). All FCD-Furin hydrogen bonds observed in simulations are summarized in Table S23 to S26

The HBond differences between different FCD sequences are all extremely statistically significant (*P* < 0.0001).

## DISCUSSION

We find weaker binding of the omicron RBD to the ACE2 as measured by H-bond counts, with mixed results for GBSA binding energy. In contrast, a number of other theory papers predict stronger ACE2-RBD binding for omicron (37 – 42), but a free energy (alchemical) perturbation analysis of the bound structure predicts weaker binding (43). The free energy perturbation analysis shares with our work a simulation starting with the observed ACE2-RBD WT structure followed by mutations. In contrast, the other theory approaches separately relax the RBD with mutations and utilize other approaches like docking (38) to bind to the ACE2.

In the Supplementary Figure, we display the correlation between interfacial RBD-ACE2 H-bond counts and GBSA binding energy for the variants included here as well as six additional variants. The correlation excluding the BA.1 and BA.2 variants is strong, with an $R^{2}$ coefficient of 0.85. The high GBSA binding energies for BA.1 and BA.2 suggest an overestimate of binding in the approach, with the largest single contribution at the Q493R residue which contributes −12.7 kcal/mol for BA.2 vs −5.3 kcal/mol for the WT RBD. Given our experience of strong correlations of H-bond counts with GBSA energies for antibody and furin binding as well, we believe this does represent an overestimate of binding free energy for the omicron variants.

Since the first posting of our work, a number of experimental papers have emerged demonstrating explicitly weaker binding of omicron RBD to ACE2 (44), weaker RBD binding and fusogenicity (consistent with weaker furin cleavage) (45, 46), and weaker expression in lung tissues [though stronger in bronchial tissue (47)]. These offer support for the predictions here. A surprise from fusogenicity studies, which reflect directly on the furin mediated cleavage at the FCD, is that omicron is 5–10 times weaker than WT or delta at yielding syncytia (45, 46). If there is a kinetic competition between sequence binding involving the N679K and P681H mutation to get the fifth residue into the deep furin pocket, there could be a strongly reduced cleavage and fusogenicity.

On the other hand, a study examining furin mediated cleavage directly on larger peptides than those considered here found that omicron led to more rapid furin cleavage than WT or delta and that this was associated with the N679K mutation as the differences largely vanished between the three variants with this mutation (48). Elsewhere, we have shown that the longer peptides can bind in a reverse orientation, and this rationalizes the difference between the variants (32). Clarification will come with more experimental studies, including Furin-FCD binding studies on the minimal six residue peptides considered here.

The binding strength of Furin to the FCD appears to correlate well with the fluctuations of the initial residue at 681. The lower the fluctuation of the backbone carbon, the lower the fluctuation of the backbone carbon for residue 685, which dominates the bonding to the furin. The P681R mutation provides the lowest C α RMSF observed among the four FCD examples considered here, and the alternate K679 starting point for omicron provides the largest C α RMSF.

The lower severity of omicron versus delta may be related to the Furin Cleavage Domain. It has been shown that this insertion is critical to the higher transmissibility of SARS-CoV-2 (6, 49) over SARS-CoV-1 and that the mutations P681H for the alpha and omicron variants and P681R for the delta variant play a large role in increased transmissibility of the variants over the wild type (7). After initial binding to the human ACE2 protein, Furin protease cleavage breaks the spike to facilitate cell wall fusion (7) and viral reproduction. The stronger the Furin-FCD H-bond binding, the more efficient the fusion at the molecular level, and ultimately, higher the viral load on the host. If the omicron acquired the P681R mutation over the P681H one, the combined antibody escape and enhanced fusion would be highly concerning.

We note that furin is not the only human enzyme that plays a role in spike cleavage and potential pathogenicity of the virus. Notably, inefficient binding of omicron to the TMPRSS2 compared to delta appears to explain the lower fusogenicity of omicron in lung epithelial cells while having comparable replication in upper respiratory cells that do not express TMPRSS2 (50). It has also been shown that the metalloprotein enzyme ADAM10, which is expressed in lung tissues, facilitates syncytia formation (51).

From an evolutionary perspective, deep mutational scan data for every point mutation of the RBD shows that few mutations lead to enhanced binding, and for the ones that do the effect is modest, while reduced binding by mutation can be dramatic (52). This suggests that ACE2 binding is already near optimal for the WT RBD. The huge number of RBD mutations that affect antibody escape for omicron inevitably drive the virus away from this optimal binding. Similarly, for the FCD, we find here and elsewhere that the binding is near optimal for the delta variant (32). Other mutants are more likely to be suboptimal or deleterious to fusion as has been observed.

In summary, a consistent picture of omicron in comparison to the delta strain is emerging. Hospitalization data points to higher disease transmissibility but lower severity for the omicron strain compared to delta (53). Our simulations see lower interfacial H-bond counts for omicron for known RBD and NTD binding regions consistent with this, as well as weaker ACE2 binding and furin binding than the delta variant. Against an immunity background tuned to the delta variant, omicron variants are more transmissible, and subsequent mutations in BA.2 and BA.5 will lead to higher transmissibility against an omicron (BA.1) tuned immunity background. Experimental studies of the binding of the RBD to ACE2 and the correlation of fusogenicity with furin binding offer support for these predictions as noted above, but more direct experiments are necessary to confirm the predictions here.

## SUPPLEMENTAL MATERIAL

Supplemental Material spectrum.01213-22-s0001.pdf Hbond counts, GBSA binding energies, HBond-GBSA binding energy correlation, and specific average HBond tables.

ASM does not own the copyrights to Supplemental Material that may be linked to, or accessed through, an article. The authors have granted ASM a non-exclusive, world-wide license to publish the Supplemental Material files. Please contact the corresponding author directly for reuse.
