# Supplementary material for: SARS-CoV-2 omicron spike simulations: broad antibody escape, weakened ACE2 binding, and modest furin cleavage
Source: Microbiol Spectr. 2023 Aug 31;11(5):e01213-22. doi: 10.1128/spectrum.01213-22 (PMC10580870; doi:10.1128/spectrum.01213-22)
Supplement: Supplemental Material — Hbond counts, GBSA binding energies, HBond-GBSA binding energy correlation, and specific average HBond tables. [file spectrum.01213-22-s0001.pdf]

Supplemental Information for:

SARS-CoV-2 omicron spike simulations: broad antibody escape, weakened ACE2 binding, modest Furin cleavage

M.Z. Jawaid, A. Baidya, R. Mahboubi Ardakani, R. L. Davis, D. L. Cox[1]

[1]Author for corresponsdence

This PDF includes:

Supplementary Data Tables

Supplementary Figure

All PDB files for simulations and AlphaFold files for the structures here can be found in the link

https://drive.google.com/drive/folders/1d3H4hA4j6L4ICdXrzrswhkz0F2z0ak2W?usp=sharing

Supplemental Data Tables

	NTD (1-300)	RBD (300-540)	FCD (679-685)
Delta	T19R, G142D, E156G, Δ157-158	L452R, T478K	P681R
Omicron BA1	A67V, Δ69-70, T95I, G142D, N211I, Δ212,ins214R	G339D, R346K, S371L, S373P, S375F, K417N, N440K, G446S, S477N, T478K, E484A, Q493R, G496S, Q498R, N501Y, Y505H	N679K, P681H
Omicron BA2	T19I, L24S, Δ25-27, G142D, V213G	G339D, R346K, S371F, S373P, S375F, T376A, D405N, K417N, N440K, S477N, T478K, E484A, Q493R, Q498R, N501Y, Y505H	N679K, P681H

Supplementary Table 1: Spike Protein Mutations relative to WT (Wuhan-Hu-1) in the N-terminal domain (NTD), receptor binding domain (RBD), and the Furin Cleavage Domain (FCD)[1].

[1]Gangavarapu, K., Latif, A. A., Mullen, J. L., Alkuzweny, M., Hufbauer, E., Tsueng, G., Haag, E., Zeller, M., Aceves, C. M., Zaiets, K., Cano, M., Zhou, J., Qian, Z., Sattler, R., Matteson, N. L., Levy, J. I., Lee, R. T., Freitas, L., … Maurer-Stroh, S. (2022). Outbreak.info genomic reports: scalable and dynamic surveillance of SARS-CoV-2 variants and mutations. Cold Spring Harbor Laboratory. https://doi.org/10.1101/2022.01.27.22269965

**Bound Pair**	**WT**	**Delta**	**BA1**	**BA2**
**RBD-ACE2**	8.71 ± 2.03	**10.57 ± 1.46**	**7.17 ± 1.48**	**7.08 ± 1.47**
**RBD-C12.B1A**	**19.23 ± 1.87**	**14.94 ± 2.61**	**11.27 ± 1.71**	**11.33 ± 1.81**
**RBD-P4A1**	**17.76 ± 2.01**	**17.61 ± 2.03**	**12.17 ± 2.45**	**14.06 ± 2.07**
**RBD-CR3022**	**12.58 ± 1.82**	**12.22 ± 1.59**	**10.81 ± 1.97**	**11.31 ± 1.45**
**NTD-4A8**	**9.62 ± 1.78**	**7.48 ± 1.49**	**4.84 ± 1.63**	**7.84 ± 2.47**
**FCD-Furin**	10.73 ± 1.73	**15.36 1.88**	**12.05 ± 1.68**	**8.65 (Alt) ± 1.59**

Supplementary Table 2: Interfacial hydrogen bonds (with standard deviations) between proteins for WT, delta, and omicron**.**

Bound Pair	WT	Delta	BA1	BA2
**RBD-ACE2**	-67.42 ± 7.47	**-72.10 ± 4.91**	**-73.97 ± 6.81**	**-78.64 ± 6.39**
**RBD-C12.B1A**	**-77.9 ± 8.00**	**-70.31 ± 8.41**	**-47.62 ± 5.78**	**-57.64 ± 6.29**
**RBD-P4A1**	**-115.91 ± 7.01**	**-110.21 ± 7.51**	**-73.71 ± 5.43**	**-106.62 ± 5.43**
**RBD-CR3022**	**-95.41 ± 2.51**	**-111.70 ± 7.80**	**-82.36 ± 10.26**	**-94.46 ± 9.38**
**NTD-4A8**	**-88.9 ± 17.9**	**-81.1 ± 5.7**	**-65.2 ± 12.2**	**-93.67 ± 6.33**
**FCD-Furin**	-83.6 ± 8.4	**-117.3 ± 4.8**	**-93.2 ± 3.8**	**-63.5 (Alt) ± 3.6**

Supplementary Table 3: MM/GBSA Binding Energies (with standard deviations) in kcal/mol between proteins for WT, delta, and omicron**.**

Supplemental Figure 1 – Correlation of HBonds and Binding Free energy

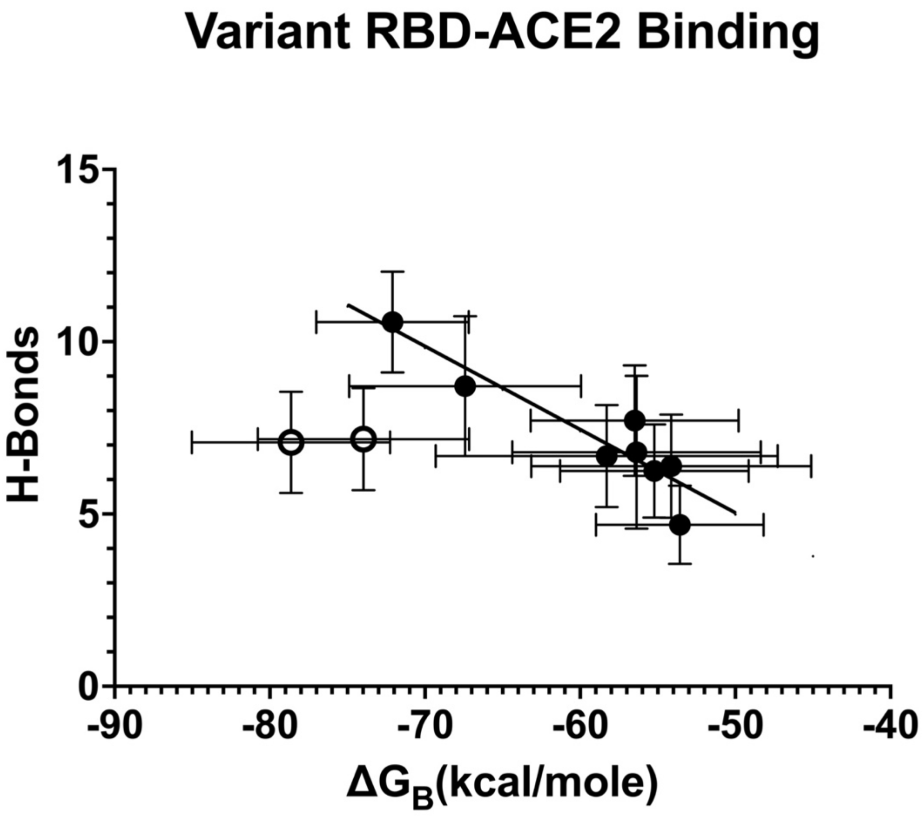

The solid circles are from simulations (left to right) Delta RBD-ACE1, WT RBD-ACE2, N439K RBD-ACE2, L452R RBD-ACE2, N501T RBD-ACE2, Beta RBD-ACE2, V367F RBD-ACE2, Alpha RBD-ACE2.

The solid line is a regression fit to those 8 points ($H - Bonds\ = -0.2415\ \Delta G_B - 7.09$ which has a regression coefficient $R^2 = 0.85$. Open circles are BA.2 (left) and BA.1 (right)

Detailed list of HBond pairs

The following tables show Hbond pairs and their detection ratios for our simulations. The Hbond pairs are counted using a distance and angle approximation as described in methods.

The bond detection ratio is defined as n/N, where n is the number of snapshots in which the labelled Hbond is detected, and N is the total number of snapshots. Therefore, a bond detection ratio of 1 means that the corresponding bond was detected in every single snapshot of the simulation.

**WT RBD**	**ACE2**	Bond detection ratio
A475	Q24.A	0.0625
	S19.A	0.596
G446	Q42.A	0.187
G476	S19.A	0.005
G496	K353.A	0.927
G502	K353.A	0.980
K417	D30.A	0.384
	H34.A	0.221
N487	Q24.A	0.557
	Y83.A	0.980
N501	K353.A	0.052
	Y41.A	0.129
Q493	E35.A	0.855
	H34.A	0.043
	K31.A	0.543
Q498	D38.A	0.293
	K353.A	0.596
	Q42.A	0.028
R403	H34.A	0.004
S477	Q24.A	0.005
	S19.A	0.139
T500	D355.A	0.365
	N330.A	0.019
	Y41.A	0.317
Y449	D38.A	0.05
	Q42.A	0.21
Y505	E37.A	0.36
	R393.A	0.01

Supplementary Table 4: HBond Pairs for WT RBD-ACE2 simulation**.**

Delta RBD	ACE2	Bond Detection Ratio
A475	Q24	0.095
	S19	0.754
G446	Q42	0.498
G496	K353	0.706
G502	K353	0.948
K417	D30	0.806
N487	Q24	0.507
	Y83	0.915
N501	K353	0.014
	Y41	0.076
Q493	E35	0.768
	H34	0.024
	K31	0.445
Q498	D38	0.028
	K353	0.735
	Q42	0.009
S477	S19	0.005
T500	D355	0.27
	N330	0.062
	Y41	0.502
Y449	D38	0.924
	Q42	0.379
Y453	H34	0.066
Y505	E37	0.829
	R393	0.043

Supplementary Table 5: HBond Pairs for Delta RBD-ACE2 simulation**.**

Omicron BA1 RBD	ACE2	Bond detection ratio
A475	Q24	0.037
	S19	0.893
G502	K353	0.968
H505	K353	0.068
N417	H34	0.006
N477	Q24	0.025
	S19	0.993
N487	Q24	0.262
	Y83	0.875
R403	H34	0.012
R493	E35	1.281
	H34	0.225
	K31	0.006
R498	Q42	0.056
S496	D38	0.862
	K353	0.012
T500	D355	0.443
	N330	0.112
	Y41	0.206
Y449	D38	0.212
Y453	H34	0.031
Y501	D38	0.019

Supplementary Table 6: HBond Pairs for Omicron BA1 RBD-ACE2 simulation**.**

Omicron BA2 RBD	ACE2	Bond detection ratio
A475	Q24	0.074
	S19	0.880
G502	K353	0.955
H519	D615	0.004
N477	Q24	0.024
	S19	0.885
N487	Q24	0.333
	Y83	0.910
R493	D38	1.686
	E35	0.766
	H34	0.024
	K31	0.004
R498	Q42	0.248
T500	D355	0.631
	N330	0.069
	Y41	0.134
Y453	H34	0.084
Y473	E23	0.004
Y489	Q24	0.004
Y501	K353	0.064

Supplementary Table 7: HBond Pairs for Omicron BA2 RBD-ACE2 simulation**.**

**WT RBD**	C1A-B12	**Bond detection ratio**
**A475**	N32.H	**0.911**
	T28.H	**0.65**
**D420**	Y58.H	**0.821**
**G476**	T28.H	**0.228**
**G496**	S30.L	**0.553**
**G502**	G28.L	**0.894**
**K417**	D96.H	**0.992**
	S98.H	**0.667**
	Y52.H	**0.691**
**K458**	S30.H	**0.041**
	S31.H	**0.098**
**L455**	Y33.H	**0.943**
**N460**	G54.H	**0.065**
**N487**	G26.H	**0.496**
	R94.H	**1.049**
**N501**	S30.L	**0.211**
**Q493**	R100A.H	**0.935**
	Y100B.H	**0.016**
**Q498**	S30.L	**0.268**
	S67.L	**0.472**
**R403**	I92.L	**1.398**
	S93.L	**0.008**
**R457**	S53.H	**0.951**
**S477**	G26.H	**0.065**
**S494**	R100A.H	**1.659**
	Y32.L	**0.041**
**T415**	Y58.H	**0.041**
**T500**	G28.L	**0.024**
**V503**	Q27.L	**0.138**
**Y421**	G54.H	**0.374**
**Y453**	G99.H	**0.967**
	Y32.L	**0.878**
**Y473**	S31.H	**0.846**
	S53.H	**0.008**
**Y489**	R94.H	**0.024**
**Y505**	D1 .L	**0.041**
	S93.L	**0.528**

Supplementary Table 8: HBond Pairs for WT RBD-C1A-B12 (7KFV) antibody simulation**.** Here, ‘H’ are residues from the heavy chain and ‘L’ are residues from the light chain.

Delta RBD	C1A-B12	Bond detection ratio
**A475**	**N32.H**	0.65
	**R94.H**	0.056
**G496**	**S30.L**	0.427
**G502**	**G28.L**	0.517
**K417**	**D96.H**	0.979
	**S98.H**	0.566
	**Y52.H**	0.678
**K458**	**S30.H**	0.014
	**S31.H**	0.014
**L455**	**Y33.H**	0.881
**N460**	**G54.H**	0.664
**N487**	**R94.H**	1.552
	**Y102.H**	0.441
**N501**	**S30.L**	0.573
**Q493**	**R100A.H**	0.399
	**Y100B.H**	0.042
	**Y32.L**	0.035
**Q498**	**S30.L**	0.035
	**S67.L**	0.413
**R403**	**I92.L**	1.455
	**S98.H**	0.077
**R457**	**S53.H**	0.643
**S477**	**E 1 .H**	0.007
	**G26.H**	0.294
**S494**	**R100A.H**	0.049
	**Y32.L**	0.049
**T415**	**Y58.H**	0.028
**T500**	**G28.L**	0.182
	**Q27.L**	0.014
**Y421**	**G54.H**	0.413
	**S53.H**	0.035
**Y453**	**G99.H**	0.58
	**Y32.L**	0.252
**Y473**	**S31.H**	0.979
**Y489**	**R94.H**	0.035
**Y495**	**Y32.L**	0.322
**Y505**	**Q27.L**	0.119
	**S93.L**	0.266

Supplementary Table 9: HBond Pairs for Delta RBD-C1A-B12 (7KFV) antibody simulation**.** Here, ‘H’ are residues from the heavy chain and ‘L’ are residues from the light chain.

Omicron BA1	C1A-B12	Bond detection ratio
A475	N32.H	0.917355
	T28.H	0.066116
D405	S93.L	0.008264
G502	G28.L	0.809917
H505	Q27.L	0.082645
K478	E 1 .H	0.024793
L455	Y33.H	0.991736
N417	Y33.H	0.066116
	Y52.H	0.132231
N460	G54.H	0.438017
	G55.H	0.082645
N477	G26.H	0.760
	T28.H	0.049
N487	G26.H	0.016
	R94.H	1.636
	Y102.H	0.190
Q474	S31.H	0.008
R403	I92.L	1.677
R408	Y58.H	0.008
R457	S53.H	0.553
R493	Y100B.H	0.082
R498	S30.L	0.107
S494	R100A.H	0.082
S496	R100A.H	0.363
T415	Y58.H	0.165
V503	Q27.L	0.008
Y421	G54.H	0.719
	S53.H	0.016
	Y33.H	0.231
Y449	R100A.H	0.008
Y473	S31.H	0.933
Y489	R94.H	0.024
Y495	Y32.L	0.049
Y501	S30.L	0.008

Supplementary Table 10: HBond Pairs for Omicron BA1 RBD-C1A-B12 (7KFV) antibody simulation**.** Here, ‘H’ are residues from the heavy chain and ‘L’ are residues from the light chain.

Omicron BA2	C1A-B12	Bond detection ratio
**A475**	**N32.H**	0.908602
	**R94.H**	0.010753
	**S31.H**	0.010753
	**T28.H**	0.139
**G502**	**Q27.L**	0.752
**K458**	**S31.H**	0.032
**L455**	**Y33.H**	0.973
**N417**	**Y33.H**	0.005
**N460**	**G54.H**	0.698
**N477**	**G26.H**	0.526
**N487**	**G26.H**	0.053
	**R94.H**	1.639
	**Y102.H**	0.209
**R403**	**I92.L**	0.924
	**S98.H**	0.521
**R405**	**D 1 .L**	0.478
	**Y94.L**	0.139
**R457**	**S53.H**	0.021
**R493**	**S31.L**	0.010
	**Y32.L**	0.478
**R498**	**G28.L**	0.032
	**S30.L**	0.118
	**S67.L**	0.016
**T500**	**G28.L**	0.784
**Y421**	**G54.H**	0.424
	**S53.H**	0.091
	**Y33.H**	0.220
**Y453**	**G99.H**	0.032
**Y473**	**S31.H**	0.817
	**S53.H**	0.021
**Y495**	**Y32.L**	0.010
**Y501**	**I29.L**	0.021

Supplementary Table 11: HBond Pairs for Omicron BA2 RBD-C1A-B12 (7KFV) antibody simulation**.** Here, 'H' are residues from the heavy chain and 'L' are residues from the light chain.

WT RBD	P4A1	Bond detection ratio
**A475**	**I28.H**	0.667
	**N32.H**	0.755
**D420**	**S56.H**	0.892
**G496**	**S30.C**	0.873
**G502**	**G28.C**	0.598
**K417**	**E101.H**	0.039
	**Q100.H**	0.912
**K458**	**S30.H**	0.176
	**S31.H**	0.52
**L455**	**Y33.H**	0.824
**N460**	**G54.H**	0.422
**N487**	**G26.H**	0.461
	**R97.H**	1.48
**N501**	**S30.C**	1.167
**Q493**	**E101.H**	0.647
**Q498**	**S67.C**	0.569
**R403**	**N92.C**	1.843
**R457**	**S53.H**	0.196
**S477**	**G26.H**	0.343
**T500**	**G28.C**	0.01
**V503**	**Q27.C**	0.01
**Y421**	**G54.H**	0.667
**Y453**	**E101.H**	0.882
**Y473**	**S31.H**	0.853
**Y489**	**R97.H**	0.049
**Y495**	**W32.C**	0.843
**Y505**	**S93.C**	1

Supplementary Table 12: HBond Pairs for WT RBD-P4A1 (7CJF) antibody simulation**.** Here, ‘H’ are residues from the heavy chain and ‘L’ are residues from the light chain.

Delta RBD	P4A1	Bond detection ratio
**A475**	**I28.H**	0.725
	**N32.H**	0.804
**D420**	**S56.H**	0.912
**G496**	**S30.L**	0.922
**G502**	**G28.L**	0.627
**K417**	**E101.H**	0.363
	**Q100.H**	0.951
**K458**	**S30.H**	0.186
	**S31.H**	0.422
	**S53.H**	0.02
**L455**	**Y33.H**	0.745
**N460**	**G54.H**	0.402
**N487**	**G26.H**	0.5
	**R97.H**	1.52
**N501**	**S30.L**	1.343
**Q493**	**E101.H**	0.167
**Q498**	**S67.L**	0.529
**R403**	**N92.L**	1.794
**R457**	**S53.H**	0.265
**S477**	**G26.H**	0.265
**V503**	**Q27.L**	0.01
**Y421**	**G54.H**	0.559
	**S53.H**	0.039
**Y449**	**S31.L**	0.01
**Y453**	**E101.H**	0.853
**Y473**	**S31.H**	0.814
**Y489**	**R97.H**	0.029
**Y495**	**W32.L**	0.843
**Y505**	**S93.L**	1

Supplementary Table 13: HBond Pairs for Delta RBD-P4A1 (7CJF) antibody simulation**.** Here, 'H' are residues from the heavy chain and 'L' are residues from the light chain.

Omicron BA1 RBD	P4A1 (7CJF)	Bond detection ratio
**A475**	**I28.H**	0.790
	**N32.H**	0.856
**D420**	**S56.H**	0.950
**G416**	**S56.H**	0.011
**G502**	**G28.L**	0.093
	**S30.L**	0.016
**H505**	**G28.L**	0.027
	**W32.L**	0.254
**K458**	**S30.H**	0.082
	**S31.H**	0.121
	**S53.H**	0.005
**L455**	**Y33.H**	0.022
**N417**	**Q100.H**	0.723
	**Y33.H**	0.961
**N460**	**G54.H**	0.232
	**G55.H**	0.027
	**S56.H**	0.055
**N477**	**G26.H**	0.950
**N487**	**R97.H**	1.419
**Q474**	**S31.H**	0.027
**R403**	**N92.L**	0.939
**R457**	**S53.H**	0.718
**R493**	**E101.H**	0.933
**R498**	**S30.L**	0.027
	**S67.L**	0.033
**T500**	**S30.L**	0.027
**V503**	**Q27.L**	0.005
**Y421**	**G54.H**	0.403
	**S53.H**	0.033
	**Y33.H**	0.480
**Y453**	**E101.H**	0.624
**Y473**	**S31.H**	0.453
**Y489**	**R97.H**	0.071
**Y501**	**W32.L**	0.071

Supplementary Table 14: HBond Pairs for Omicron BA1 RBD-P4A1 (7CJF) antibody simulation**.** Here, 'H' are residues from the heavy chain and 'L' are residues from the light chain.

Omicron BA2 RBD	P4A1 (7CJF)	Bond detection ratio
**A475**	**I28.H**	0.678218
	**N32.H**	0.816832
**A520**	**T393**	0.00495
**D420**	**S56.H**	0.886139
**E516 ND**	**N394**	0.00495
**G502**	**G28.L**	0.985
**H505**	**N92.L**	0.044
	**Q27.L**	0.128
**K458**	**S30.H**	0.019
	**S31.H**	0.123
**L455**	**Y33.H**	0.079
**N405**	**N92.L**	0.108
**N417**	**Q100.H**	0.821
	**Y33.H**	0.905
	**Y52.H**	0.039
**N460**	**G54.H**	0.311
	**S56.H**	0.029
**N477**	**F27.H**	0.004
	**G26.H**	0.514
**N487**	**G26.H**	0.034
	**R97.H**	1.569
	**Y107.H**	0.005
**Q474**	**S31.H**	0.005
**R403**	**E101.H**	1.524
	**N92.L**	0.113
**R457**	**S53.H**	0.272
**R493**	**E101.H**	0.915
**T500**	**S30.L**	0.292
**Y421**	**G54.H**	0.658
	**S53.H**	0.019
	**Y33.H**	0.084
**Y453**	**E101.H**	0.099
**Y473**	**S31.H**	0.698
	**S53.H**	0.064
**Y489**	**R97.H**	0.054
**Y501**	**S31.L**	0.727

Supplementary Table 15: HBond Pairs for Omicron BA2 RBD-P4A1 (7CJF) antibody simulation**.** Here, ‘H’ are residues from the heavy chain and ‘L’ are residues from the light chain.

**WT RBD**	**CR3022**	**Bond detection ratio**
**C379**	**I102.H**	0.932
**D428**	**S32.L**	1.429
	**S33.L**	0.91
	**Y31.L**	0.023
**F374**	**K74.H**	0.008
**F377**	**Y52.H**	0.932
**G381**	**I102.H**	0.91
	**Y38.L**	0.985
**H519**	**N35.L**	0.098
**K378**	**D55.H**	0.902
	**E57.H**	0.857
**K386**	**D107.H**	0.895
	**E61.L**	0.812
**N370**	**Y27.H**	0.707
**S366**	**Y27.H**	0.008
**S383**	**S100.H**	0.511
	**T104.H**	0.241
**T376**	**D55.H**	0.218
**T385**	**Q 1 .H**	0.008
	**S100.H**	0.902
	**Y32.H**	0.045
**T430**	**S33.L**	0.143
	**Y31.L**	0.113

Supplementary Table 16: HBond Pairs for Omicron BA1 RBD-CR3022 (6YOR) antibody simulation**.** Here, ‘H’ are residues from the heavy chain and ‘L’ are residues from the light chain.

Delta RBD	CR3022	Bond Detection Ratio
**C379.E**	**I102.H**	0.935
**D389.E**	**T59.L**	0.153
**D428.E**	**S32.L**	0.089
	**Y31.L**	0.048
**F374.E**	**K74.H**	0.008
**F377.E**	**Y52.H**	0.96
**G381.E**	**I102.H**	0.935
	**T104.H**	0.008
	**Y38.L**	0.984
**H519.E**	**N35.L**	0.032
**K378.E**	**D55.H**	0.815
	**E57.H**	0.855
**K386.E**	**D107.H**	0.758
	**E61.L**	0.871
	**Y55.L**	0.048
**L517.E**	**N35.L**	0.919
	**S33.L**	0.863
**N370.E**	**G28.H**	0.185
**Q414.E**	**E57.H**	0.008
**S375.E**	**K74.H**	0.04
**S383.E**	**S100.H**	0.331
	**T104.H**	0.089
**T376.E**	**D55.H**	0.016
**T385.E**	**D107.H**	0.056
	**Q 1 .H**	0.081
	**S100.H**	0.524
**T430.E**	**S33.L**	0.927
**V382.E**	**T104.H**	0.032
**Y369.E**	**Q 1 .H**	0.065
**Y380.E**	**E57.H**	0.137

Supplementary Table 17: HBond Pairs for Delta RBD-CR3022 (6YOR) antibody simulation**.** Here, 'H' are residues from the heavy chain and 'L' are residues from the light chain.

Omicron BA1	CR_3022 (6YOR)	Bond detection ratio
C379	I102.H	0.971
D428	S32.L	1.482
	S33.L	0.352
	Y31.L	0.115
F377	Y52.H	0.949
G381	I102.H	0.719
	Y38.L	0.812
H519	N35.L	0.115
K378	D55.H	0.834
	E57.H	0.784
K386	D107.H	0.748
	E61.L	0.755
	Q 1 .H	0.007
L517	N35.L	0.007
N370	G28.H	0.014
	Y27.H	0.244
N388	Q 1 .H	0.021
	Y55.L	0.007
S366	Y27.H	0.165
S383	S100.H	0.899
	T104.H	0.028
T376	D55.H	0.187
T385	Q 1 .H	0.100
	S100.H	0.460
	T31.H	0.064
	Y32.H	0.035
T430	S33.L	0.071

Supplementary Table 18: HBond Pairs for Omicron BA1 RBD-CR_3022 (6YOR) antibody simulation**.** Here, ‘H’ are residues from the heavy chain and ‘L’ are residues from the light chain.

**Omicron BA2**	**CR_3022 (6YOR)**	**Bond detection Ratio**
**C379**	**I102.H**	**0.968**
**D428**	**S32.L**	**0.819**
**F375**	**K74.H**	**0.021**
**F377**	**Y52.H**	**0.936**
**F515**	**S33.L**	**0.010**
**G381**	**I102.H**	**0.856**
	**Y38.L**	**0.984**
**H519**	**N35.L**	**0.015**
**K378**	**D55.H**	**0.856**
	**E57.H**	**0.691**
**K386**	**D107.H**	**0.898**
	**E61.L**	**0.824**
**L517**	**N35.L**	**0.632**
	**S33.L**	**0.989**
**N370**	**Y27.H**	**0.25**
**N388**	**Y55.L**	**0.015**
**S366**	**Y27.H**	**0.164**
**S383**	**S100.H**	**0.872**
**T385**	**S100.H**	**0.005**
	**Y32.H**	**0.010**
**T430**	**S33.L**	**0.425**

Supplementary Table 19: HBond Pairs for Omicron BA2 RBD-CR_3022 (6YOR) antibody simulation**.** Here, ‘H’ are residues from the heavy chain and ‘L’ are residues from the light chain.

WT NTD	4A8	Bond Detection Ratio
K147	E72.H	0.703
	L29.H	0.673
	L32.H	0.446
K150	D55.H	0.03
	E54.H	0.079
	P53.H	0.01
	Y111.H	0.04
K97	D107.H	0.099
L249	A103.H	0.05
	V102.H	0.693
N149	D55.H	0.465
	E54.H	0.069
	P53.H	0.663
N99	P106.H	0.366
R246	E31.H	0.02
	G26.H	1.396
S247	Y27.H	0.614
S254	E 1 .H	0.158
S98	D107.H	0.228
T250	T105.H	0.01
W152	Y111.H	0.307
Y145	E31.H	0.327
	T30.H	0.743
Y248	E31.H	0.99

Supplementary Table 20: HBond Pairs for WT NTD-4A8 (7C2L) antibody simulation**.** Here, 'H' are residues from the heavy chain and 'L' are residues from the light chain.

Delta NTD	4A8	Bond detection ratio
A292	D290	0.017
D290	R273	0.017
D294	S297	0.05
H146	T30.H	0.008
K147	E72.H	0.125
	L29.H	0.45
	L32.H	0.583
	T30.H	0.025
K150	D55.H	0.008
	E54.H	0.6
	P53.H	0.033
	Y111.H	0.017
N148	D55.H	0.167
N149	D55.H	0.708
P295	T299	0.042
Q14	E 1 .H	0.117
	G26.H	0.183
	S75.H	0.017
	T28.H	0.025
R246	E31.H	1.8
S247	G104.H	0.008
S254	E 1 .H	0.008
	Y27.H	0.008
S297	C291	0.008
S297	D294	0.183
W258	E 1 .H	0.017
Y145	Y111.H	0.05
Y248	E31.H	0.917
	G104.H	1.483

Supplementary Table 21: HBond Pairs for Delta NTD-4A8 (7C2L) antibody simulation**.** Here, ‘H’ are residues from the heavy chain and ‘L’ are residues from the light chain.

BA1 NTD	4A8	Bond Detection Ratio
K147	E72.H	0.540
	L29.H	0.338
	L32.H	0.048
	S33.H	0.008
K150	E54.H	0.169
	P53.H	0.016
	Y113.H	0.008
K97	D107.H	0.169
L249	V102.H	0.943
N149	D55.H	0.096
	E54.H	0.048
	P53.H	0.669
N99	D107.H	0.016
	P106.H	0.419
R246	E31.H	0.169
	G26.H	0.056
	T100.H	0.008
	Y27.H	0.016
S247	Y27.H	0.016
S254	N58.L	0.032
	Y54.L	0.024
S98	D107.H	0.217
	P106.H	0.056
T250	A103.H	0.088
	G104.H	0.161
W152	D107.H	0.008
	T105.H	0.153
	Y111.H	0.008
Y248	E31.H	0.088

Supplementary Table 22: HBond Pairs for Omicron BA1 NTD-4A8 (7C2L) antibody simulation**.** Here, ‘H’ are residues from the heavy chain and ‘L’ are residues from the light chain.

BA2 NTD	4A8	Bond Detection Ratio
K147.A	E72.H	0.675
	L29.H	0.682
	L32.H	0.299
K150.A	E54.H	0.274
	P53.H	0.21
	Y111.H	0.013
K97.A	D107.H	0.038
L249.A	G104.H	0.389
	V102.H	0.395
N148.A	D55.H	0.006
N149.A	D55.H	0.471
	E54.H	0.013
	P53.H	0.408
N99.A	D107.H	0.019
	P106.H	0.076
R246.A	E31.H	0.115
	G26.H	0.503
	T28.H	0.013
S247.A	Y27.H	0.471
S254.A	E 1 .H	0.459
	S61.L	0.07
S98.A	D107.H	0.153
W152.A	Y111.H	0.115
Y145.A	E31.H	0.363
	T30.H	0.841
Y248.A	E31.H	1
	G104.H	0.146
	Y27.H	0.019

Supplementary Table 23: HBond Pairs for Omicron BA2 NTD-4A8 (7C2L) antibody simulation**.** Here, ‘H’ are residues from the heavy chain and ‘L’ are residues from the light chain.

FCD	Furin	Bond detection ratio
P1	E123	0.026
	E150	0.043
	V124	0.184
R2	D157	1.473
	E129	0.885
	G158	0.061
	Y201	0.745
R3	D151	0.228
	E150	0.921
	G148	1.771
R5	A185	0.921
	D151	0.017
	D199	1.263
	P149	0.087
	S146	0.798
	S261	1
S6	H87	0.315
	N188	0.324
	S261	0.140
S6	H87	0.008
S6	N188	0.008

Supplementary Table 24: HBond Pairs for WT (PRRARS) FCD-Furin simulation**.**

Delta FCD	Furin	Bond detection ratio
R1	D152	0.309
	D157	0.097
	E123	0.085
	E150	0.036
	G122	0.012
	T155	0.533
R2	D157	1.412
	E129	0.891
	G158	0.006
	Y201	0.8
R3	E150	1.648
	G148	1.733
R5	A185	0.909
	D151	1.867
	D199	1.442
	S146	0.964
	S261	0.382
	D199	0.006
S6	H87	0.406
	N188	0.606
	S261	0.564
	T258	0.242
S6	H87	0.018
S6	S261	0.018
S6	N188	0.018

Supplementary Table 25: HBond Pairs for Delta (RRRARS) FCD-Furin simulation**.**

BA1, BA2 FCD (HRRARS)	Furin	Bond detection ratio
H1	E123	0.05
	E150	0.007
	G122	0.021
R2	D157	1.773
	E129	0.05
	G158	0.085
	Y201	0.816
R3	D151	0.383
	E150	1.326
	G148	0.922
R5	A185	1
	D199	1.794
	P149	0.22
	S146	0.879
	S261	1.702
S6	H87	0.894
	N188	0.106
	S261	0.007

Supplementary Table 26: HBond Pairs for BA1, BA2, FCD-Furin simulation**.**

BA1, BA2 Alternate FCD (KSHRRA)	Furin	Bond detection ratio
A6	N188	0.583
H3	E150	0.259
	G148	1.878
K1	D121	0.094
	E123	0.554
	E150	0.518
	G122	0.014
	V124	0.072
R4	D46	1.712
	H87	0.101
	S261	0.029
R5	A185	0.022
	D151	0.014
	D199	1.504
	S146	0.849
	S186	0.058
	S261	0.05
	T202	0.05
	T260	0.007
	Y201	0.007
S2	E129	0.108
	V124	0.014

Supplementary Table 27: HBond Pairs for alternate BA1, BA2, FCD-Furin simulation**.**
